# Predicting Type 2 Diabetes Using Logistic Regression and Machine Learning Approaches

**DOI:** 10.3390/ijerph18147346

**Published:** 2021-07-09

**Authors:** Ram D. Joshi, Chandra K. Dhakal

**Affiliations:** 1 Department of Economics, Texas Tech University, Lubbock, TX 79409, USA; ram.d.joshi@ttu.edu; 2 Department of Agricultural and Applied Economics, University of Georgia, Athens, GA 30602, USA

**Keywords:** decision tree, diabetes risk factors, machine learning, prediction accuracy

## Abstract

Diabetes mellitus is one of the most common human diseases worldwide and may cause several health-related complications. It is responsible for considerable morbidity, mortality, and economic loss. A timely diagnosis and prediction of this disease could provide patients with an opportunity to take the appropriate preventive and treatment strategies. To improve the understanding of risk factors, we predict type 2 diabetes for Pima Indian women utilizing a logistic regression model and decision tree—a machine learning algorithm. Our analysis finds five main predictors of type 2 diabetes: glucose, pregnancy, body mass index (BMI), diabetes pedigree function, and age. We further explore a classification tree to complement and validate our analysis. The six-fold classification tree indicates glucose, BMI, and age are important factors, while the ten-node tree implies glucose, BMI, pregnancy, diabetes pedigree function, and age as the significant predictors. Our preferred specification yields a prediction accuracy of 78.26% and a cross-validation error rate of 21.74%. We argue that our model can be applied to make a reasonable prediction of type 2 diabetes, and could potentially be used to complement existing preventive measures to curb the incidence of diabetes and reduce associated costs.

## 1. Introduction

Diabetes is one of the most common human diseases and has become a significant public health concern worldwide. There were approximately 450 million people diagnosed with diabetes that resulted in around 1.37 million deaths globally in 2017 [1]. More than 100 million US adults live with diabetes, and diabetes was the seventh leading cause of death in the US in 2020 [2]. One in ten US adults have diabetes now, and if the current trend continues, it is projected that as many as one in three US adults could have diabetes by 2050 [2]. Diabetes patients are at elevated risk of developing health complications such as kidney failure, vision loss, heart disease, stroke, premature death, and amputation of feet or legs, which can lead to dysfunction and chronic damage of tissue [3]. In addition, there are substantial economic costs associated with the disease. The total estimated price of diagnosed diabetes in the US increased to USD 237 billion in 2017 from USD 188 billion in 2012. The excess medical costs per person associated with diabetes increased to USD 9601 from USD 8417 during the same period [2]. Additionally, there could be productivity loss due to diabetic patients in the workforce.

An individual at high risk of diabetes may not be aware of the risk factors associated with it. Given the high prevalence and severity of diabetes, researchers are interested in finding the most common risk factors of diabetes, as it could be due to a combination of several reasons. Determining the risk factors and early prediction of diabetes have been vital in reducing diabetes complications [4,5] and economic burden [6] and is beneficial from both clinical practice and public health perspectives [7]. Similarly, studies find that screening high-risk individuals identifies the population groups in which implementing measures aimed at preventing diabetes will be the most beneficial [8]. Early intervention may help prevent complications and improve quality of life and are essential in designing effective prevention strategies [9,10]. There is growing evidence that lifestyle modification prevents or delays type 2 diabetes [11]. The main risk factors of diabetes are considered to be an unhealthy diet, aging, family history, ethnic groups, obesity, sedentary lifestyle, and previous history of gestational diabetes [6,7,12]. Previous studies have also reported that sex, body mass index (BMI), pregnancy, and metabolic status are associated with diabetes [13,14].

Prediction models can screen pre-diabetes or people with an increased risk of developing diabetes to help decide the best clinical management for patients. Numerous predictive equations have been suggested to model the risk factors of incident diabetes [15,16,17]. For instance, Heikes et al. [18] studied a tool to predict the risk of diabetes in the US using undiagnosed and pre-diabetes data, and Razavian et al. [19] developed logistic regression-based prediction models for type 2 diabetes occurrence. These models also help screen individuals to posit individuals who are at a high risk of having diabetes. Zou et al. [20] used machine learning methods to predict diabetes in Luzhou, China, and a five-fold cross-validation was used to validate the models. Nguyen et al. [5] predict the onset of diabetes employing deep learning algorithms suggesting that sophisticated methods may improve the performance of models. In contrast, several other studies have shown that logistic regression performs as least as well as machine learning techniques for disease risk prediction ([21,22], for example). Similarly, Anderson et al. [23] used logistic regression along with machine learning algorithms and found a higher accuracy with the logistic regression model. These are mainly based on assessing risk factors of diabetes, such as household and individual characteristics; however, the lack of an objective and unbiased evaluation is still an issue [24]. Additionally, there is growing concern that those predictive models are poorly developed due to inappropriate selection of covariates, missing data, small samples size, and wrongly specified statistical models [25,26]. To this end, only a few risk prediction models have been routinely used in clinical practice. The reliability and quality of these predictive tools and equations show significant variation depending on geography, available data, and ethnicity [5]. Risk factors for one ethnic group may not be generalized to others; for example, the prevalence of diabetes is reported to be higher among the Pima Indian community. Therefore, this study uses the Pima Indian dataset to predict if an individual is at risk of developing diabetes based on specific diagnostic factors [27,28].

We consider a combined approach of logistic regression and machine learning to predict the risk factors of type 2 diabetes mellitus. The logistic regression compares several prediction models for predicting diabetes. We used various selection criteria popular in the literature such as *AIC*, *BIC*, Mallows’ Cp, adjusted $R^{2}$, and forward and backward selection to identify the significant predictors. We then exploit the classification tree, a widely used machine learning technique with considerable classification power [5,20,25], to predict diabetes incidence in several previous studies. Our paper also contributes to the broad literature on risk factors of diabetes. We extend previous research by exploring traditional econometric models and simple machine learning algorithms to build on the literature on diabetes prediction. Our analysis finds five main predictors of diabetes: glucose, pregnancy, body mass index, age, and diabetes pedigree function. These risk factors of diabetes identified by the logistic regression were validated by the decision tree and could help classify high-risk individuals and prevent, diagnose and manage diabetes. A regression model containing the above five predictors yields a prediction accuracy of 77.73% and a cross-validation error rate of 22.65%. This study helps inform policymakers and design the health policy to curb the prevalence of diabetes. The methods exhibiting the best classification performance vary depending on the data structure; therefore, future studies should be cautious using a single model or approach for diabetes risk prediction.

The remainder of this article proceeds as follows. The next section discusses data and summary statistics. We then describe the methods. This is followed by a presentation of the results. The last section concludes our study.

## 2. Data and Summary Statistics

Several factors might be related to diabetes, including blood pressure, pregnancies for women, age and body mass index, etc. As a component of diabetes management, it would be helpful to know which variables are related to diabetes. We use the Pima Indian dataset, made available by the National Institute of Diabetes at the Johns Hopkins University, as a test case to predict the risk factors associated with diabetes. The Pima are American Indians that live along the Gila River and Salt River in Southern Arizona. Each observation in the dataset represents an individual patient and includes information on the patient’s diabetes classification, along with the various medical attributes such as the number of pregnancies, plasma glucose concentration, tricep skinfold thickness, body mass index (BMI), diastolic blood pressure, 2-hour serum insulin (serum-insulin), age and diabetes pedigree function. Our response variable, diabetes, would take the value of 1 if an individual were diagnosed with type 2 diabetes and 0 otherwise. There are 268 (34.9%) diabetes patients in our sample. Five covariates, insulin, glucose, BMI, skin thickness, and blood pressure, contain at least one missing value (indexed by zero), which is not meaningful. So, we replaced all those zeros with the corresponding median values. We analyzed data using statistical program R version 4.0.5 [29]. Table 1 presents descriptive statistics for all predictors after median value imputation for missing values.

Table 1 shows that BP, BMI, and skin-thickness have almost the same mean and median. The variable pedigree has the lowest standard deviation while insulin has the highest standard deviation, implying that pedigree has the least variability and insulin has the highest variability present in their distributions. Our objective is to identify a subset of covariates most suitable for inclusion in a predictive model for diabetes. Including only a few predictors can lead to omitted variable bias, and too many predictors can reduce the precision. There are numerous methods available for developing appropriate predictive equations. Among them, we implement logistic regression and a classification tree—a machine learning technique.

## 3. Methods

### 3.1. Logistic Regression

Logistic regressions model a relationship between the categorical response variable and covariates. Specifically, there is a linear combination of independent variables with log-odds of the probability of an event in a logistic model. Binary logistic regressions estimate the likelihood that a characteristic of a binary variable is present, given the values of the covariates. Suppose *Y* is a binary response variable where $Y_{i}=1$ if the character is present and $Y_{i}=0$ if the character is absent and the data $\left[Y_{1},Y_{2},...,Y_{n}\right]$ are independent. Let $\pi_{i}$ be the probability of success. Additionally, consider $x=(x_{1},x_{2},...,x_{p})$ as a set of explanatory variables which can be discrete, continuous, or a combination of both discrete and continuous. Then, the logistic function for $\pi_{i}$ is given by

$$logit\left(\pi_{i}\right)=log(\frac{\pi_{i}}{1-\pi_{i}})=\beta_{0}+\beta_{1}x_{i1}+\beta_{2}x_{i2}+\cdots +\beta_{p}x_{i,p};$$

where

$$\begin{array}{c} \pi_{i}=\frac{exp(\beta_{0}+\beta_{1}x_{i1}+\beta_{2}x_{i2}+\cdots +\beta_{p}x_{i,p})}{1+exp(\beta_{0}+\beta_{1}x_{i1}+\beta_{2}x_{i2}+\cdots +\beta_{p}x_{i,p})}=\frac{exp(x_{i}^{\prime }\beta )}{1+exp(x_{i}^{\prime }\beta )}=\Lambda \left(x_{i}^{\prime }\beta \right) \end{array}$$

Here, $\pi_{i}$ denotes the probability that a sample is in a given category of the dichotomous response variable, commonly called as the "success probability" and, clearly, $0\leq \pi_{i}\leq 1$. Λ(.) is the logistic cdf, with $\lambda \left(z\right)=e^{z}/\left(1+e^{-z}\right)=1/\left(1+e^{-z}\right)$ and $\beta^{s}$ represents a vector of parameters to be estimated (Cameron and Trivedi, 2005). The expression

$\left(\frac{\pi_{i}}{1-\pi_{i}}\right)$ is called the odds ratio or relative risk.

#### Estimation and Likelihood Ratio Test

Maximum likelihood is the preferred method to estimate β since it has better statistical properties, although we can use the least-squares approach. Consider, the logistic model with the single predictor variable *X* given by the logistic function of

$$\pi \left(X\right)=\frac{exp(X_{i}\beta )}{1+exp(X_{i}\beta )}$$

We wish to find the estimates such that plugging $\hat{\beta }$ into the model for π(X) gives a number close to one for all subjects who have diabetes and close to zero otherwise. Mathematically, the likelihood function is given by

$$L\left(\beta_{0},\beta_{1}\right)=\prod_{i:y_{i}=1}\pi \left(x_{i}\right)\prod_{i^{\prime }:y_{i^{\prime }}=0}\left(1-\pi \left(x_{i^{\prime }}\right)\right)$$

The estimates $\hat{\beta }$ are chosen to maximize this likelihood function. We take the logarithm on both sides to calculate and use the log-likelihood function for the estimation purpose.

We used the likelihood ratio to test if any subset of estimates β is zero. Suppose that *p* and *r* represent the number of β in the full model and the reduced model, respectively. The likelihood ratio test statistic is given by

$$\Lambda^{*}=-2[l\left({\hat{\beta }}^{\left(0\right)}\right)-l\left(\hat{\beta }\right)],$$

where $l(\hat{\beta })$ and $l({\hat{\beta }}^{\left(0\right)})$ are the log likelihoods of the full model and the reduced model, respectively, evaluated at the maximum likelihood estimation (MLE) of that reduced and $\Lambda^{*}∼\chi_{p-r}^{2};$ *p* and *r* being the number of parameters in the full and the reduced model, respectively.

### 3.2. Model Selection Criteria

We used Akaike’s information criteria (*AIC*), Schwarz’s Bayesian information criteria (*BIC*), adjusted $R^{2}$, and *PRESS* to select the best predictive model. Information criteria are procedures that attempt to choose the model with the lowest sum of squared errors (*SSE*), with penalties for including too many parameters. *AIC* estimates the relative distance between the true and fitted likelihood functions of the data and model plus a constant. The *AIC* criteria are to choose the model which yields the smallest value of *AIC*, as defined by

$$AIC_{p}=n\log\left(SSE\right)-n\log\left(n\right)+2p,$$

where *n*, and *p* number of observations and the number of parameters, respectively.

The *BIC* gives a function of the posterior probability of a true model under a certain Bayesian setup. The *BIC* criteria are to choose the model which yields the smallest value of *BIC*. We define *BIC* as

$$BIC_{p}=n\log\left(SSE\right)-n\log\left(n\right)+p\log\left(n\right),$$

where parameters are as defined earlier. Note that *BIC* incorporates a higher penalty for a higher *n*, and so it rewards more parsimonious models.

The prediction sum of squares (*PRESS*) is used to assess the model’s predictive ability and can also be used to compare regression models as a model validation method. For n observations, *PRESS* is determined by excluding each point at once, and then the remaining n−1 observation points are used to predict the value of the omitted response, denoted by ${\hat{y}}_{i(i)}$. We then calculated the $i^{th}$ *PRESS* residual as the difference $y_{i}-{\hat{y}}_{i(i)}$. The formula for *PRESS* is given by

$$PRESS=\sum_{i=1}^{n}{\left(y_{i}-{\hat{y}}_{i(i)}\right)}^{2}.$$

The smaller the *PRESS* value, the better the model’s prediction ability, which is helpful to validate the predictive ability of the model without selecting another sample or subsetting the data into training and to assess the predictive power. Predictive $R^{2}$ (denoted by $R_{Pred}^{2})$ which is more intuitive than *PRESS* itself, is defined as

$$R_{Pred}^{2}=1-\frac{PRESS}{SSTO}.$$

*PRESS* and $R_{Pred}^{2}$ together can help prevent over-fitting because both are computed using observations, not in the model estimation.

$R^{2}$ is the proportion of the variance in the dependent variable that is predictable from the independent variable(s) and is defined as

$$R^{2}=\frac{SSR}{SSTO}=1-\frac{SSE}{SSTO},$$

where *SSR* and *SSTO* are the regression sum of squares and total sum of squares, respectively. We can see that $R^{2}$ increases with an increase in predictors. Therefore, a model based on the largest $R^{2}$ value may not be the best predictive equation. Penalizing for adding more predictors to the model seems more plausible as the adjusted $R^{2}$, denoted by $R_{a}^{2}$ and defined by

$$R_{a}^{2}=1-\frac{n-1}{n-p}\frac{SSE}{SSTO}=1-\frac{n-1}{SSTO}MSE,$$

where *MSE* is defined as *MSE* $=\frac{SSE}{n-p}=\frac{\sum_{(}y_{i}-{\hat{y}}_{i}{)}^{2}}{n-p}$.

Note that *MSE* is minimal if and only if $R_{a}^{2}$ is at its highest value.

### 3.3. Validation and Cross-Validation Method

We can estimate the test error using the validation set and cross-validation error methods as an alternative to the above-described approaches. We calculated the cross-validation error and validation set error for each model under consideration and then selected the model with the lowest test error as our preferred specification. It can also be used in a broader range of model selection problems where it is hard to figure out the number of covariates in the model or complex to estimate the error variance $\sigma^{2}$.

Validation set approach: To estimate the test error rate associated with a particular method on a set of samples, we used the validation set approach that randomly divides the available samples into a training set and a validation set. The model fits the training set. We used the fitted model to predict the responses in the validation set. The resulting validation set error rate-typically assessed using mean square errors (MSEs).

k-fold cross-validation: This approach randomly divides the sample into k-groups or folds of equal size. The first fold behaves as a validation set, and the model is fitted with the remaining k-1 folds. The mean squared error, MSE, is then calculated using the samples in the held-out fold. We repeat this process k times; each time, a different group of samples behaves as a validation set [30]. This process yields k estimates of the test errors; $MSE_{1},MSE_{2},\cdots ,MSE_{k}$. The advantages of this method is that full data are trained and tested that help lowers the variance [31]. The k-fold CV estimate is determined by averaging these values:

$$CV_{\left(k\right)}=\frac{1}{k}\sum_{i=1}^{k}MSE_{i}$$

### 3.4. Classification Tree

A classification tree is a basic regression method with a tree structure that begins with a single node representing the training set. We used a classification tree to predict a qualitative response. The expected response for a sample is computed by the mean response of the training set that lies to the same terminal node. We predicted that each sample belongs to the most frequently occurring class of training sets. We are also interested in the classes among the training sets [30].

The classification error rate is the fraction of the training set in a region that does not belong to the most common class:

$$E=1-max_{k}\left({\hat{p}}_{mk}\right)\cdots \left(*\right)$$

where ${\hat{p}}_{mk}$ represents the proportion of the training set from the kth class in the mth region. Two other measures are preferable when classification error is not sensitive for tree growing.

The Gini index [32] is defined by

$$G=\sum_{k=1}^{K}{\hat{p}}_{mk}\left(1-{\hat{p}}_{mk}\right)\cdots \left(**\right)$$

and this is a measure of total variance across *K* classes. Note that the Gini index is referred to as a measure of node purity and equal to a small value if all of the ${\hat{p}}_{mk}s$ approximate to zero or one. A small value indicates that a node contains observations from a single class. Prediction accuracy is one of the most commonly used criteria in the classification tree. We also measured the performance of classifiers using the prediction accuracy, which is defined as the proportion of all subjects that were correctly predicted. The accuracy of models was calculated through the confusion matrix as

$$Accuracy=\frac{TP+TN}{TP+FP+TN+FN}\times 100\%$$

where *TP*, *TN*, *FP*, and *FN* are true positive, true negative, false positive, and false negative, respectively.

## 4. Results

**Figure 1:**
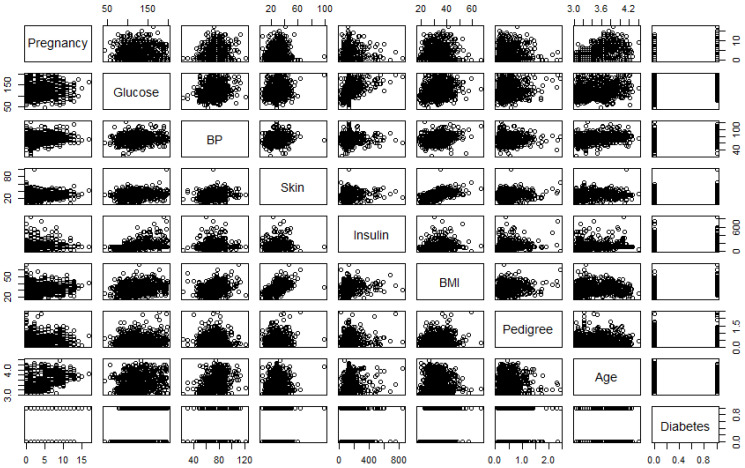
Correlation plot of the variables used in the study. Note: BP and BMI represent blood pressure and body mass index, respectively.

Figure 1 is the correlation plot, which gives the strength of the correlations between different pairs of the predictors. The pairwise correlations (*r*) between pregnancy and age (0.59) and between BMI and skin thickness (0.54) are (r>0.5) high compared to other pairs, indicating these two pairs of predictors are significantly correlated.

### 4.1. Estimates of Logistic Regression Models

We begin by looking at the predictors of the prevalence of diabetes using the logistic regression model. We tried all possible combinations of predictors and then compared each model using the goodness of fit test and model selection criteria. We outlined five candidate models while performing the goodness of fit test. Table 2 shows results from fitting the logistic regression models, in which we compared results across five different candidate regression specifications and a null model. Model 1 represents estimates from the null model, while model 2 estimates the full model. Since the residual deviance is decreased significantly with the inclusion of predictor variables, we can say that the model with the inclusion of predictor variables is better than the null model. Based on the full model, the variables skin thickness, blood pressure, and insulin are not significant predictors of diabetes at 5% significance level. Model 3 is the estimate of the logistic regression, omitting the non-significant variable from model 2 as we want to ensure a better estimation of the regression coefficients. Model 4, model 5, and model 6 add different interaction terms as we observe interacting age with pregnancy, glucose, and insulin, and insulin with pedigree provides more meaningful results.

We proceed to obtain the best possible model specified by the best subset selection method. For this purpose, we used *AIC*, *BIC*, log-likelihood, $R^{2}$, adjusted $R^{2}$, and $C_{p}$ criteria. Model 6 has the smallest *AIC* and the largest $R^{2}$ and log-likelihood values, suggesting a better fit; however, model 4 has the smallest *BIC*, indicating ambiguity. Notice that $R^{2}$ and log-likelihood values increase continuously with each additional variable included in the model. These two criteria are not much helpful for selecting the best fit model since these statistics do not indicate which and how many predictor variables to include. Additionally, we have to be cautious about the possibility of obtaining an over-fitted model while including several covariates and interaction terms that could lead to a problem with near co-linearity.

**Figure 2:**
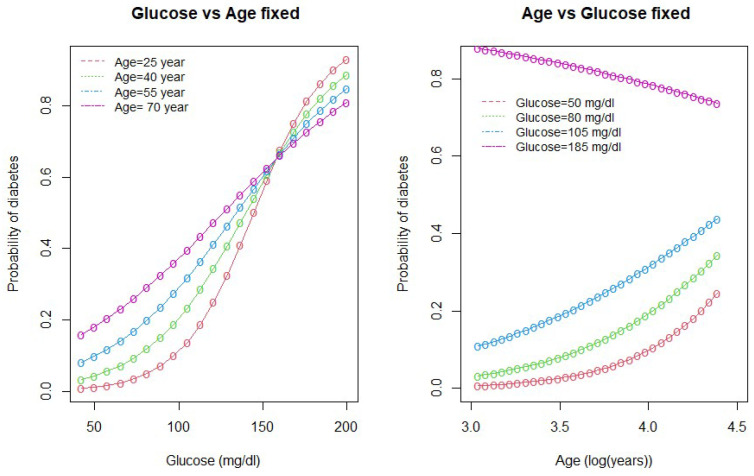
Interaction between age and glucose.

**Figure 3:**
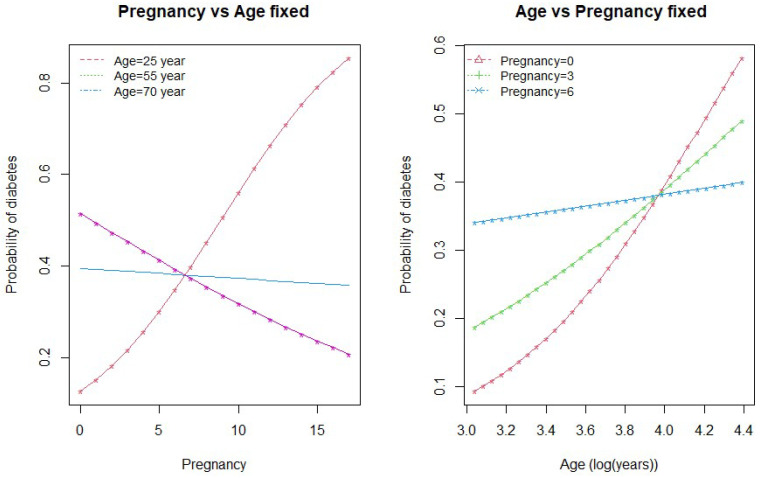
Interaction between age and pregnancy.

We drew interaction plots to better understand the nature of the interactions between age and glucose and between age and pregnancy. Figure 2 and Figure 3 show how the variation in one of the predictor variable changes the response given a fixed value of the interacting variable.

As seen in Figure 2, the probability of diabetes is higher for high-age groups when the glucose level is below 160 mg/dL (approx). However, when the glucose level is above 160 mg/dL, the probability of diabetes is higher for low-age groups and lower for high-age group women. The second plot in the Figure 2 shows that the probability of diabetes is the highest for women with high glucose levels (185 mg/dL). The lower the glucose level, the lower the probability of diabetes.

Figure 3 shows the interaction between age and pregnancy. As seen in the plot, there is an inverse relationship between age and pregnancy in the prediction of diabetes. When the number of pregnancies is greater than 7, the probability of diabetes is higher for low age groups (<55 years) and less for higher age group women. This relationship is the opposite when the number of pregnancies for a woman is less than 7. On the other hand, if we keep pregnancy fixed and vary age, the probability of diabetes is higher for the higher age group (>55 years) who have no children and the lowest for the women of age ≥55 years who have had six pregnancies. At the same time, the probability of diabetes incidence remains lower if a woman is in the low-age group with fewer than six pregnancies.

**Figure 4:**
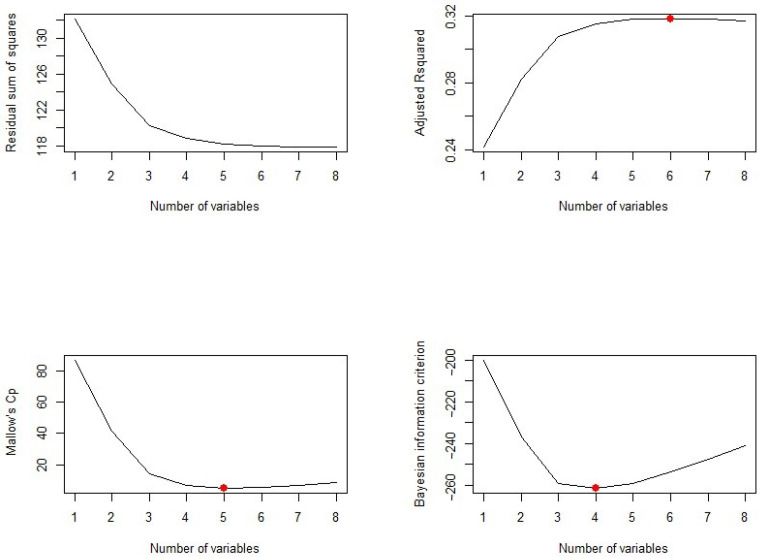
Number of significant predictors indicated by different criteria.

The plots of residual sum of square (RSS), adjusted $R^{2}$, $C_{p}$, and *BIC* for all models together will help us decide the best fit model to select. Figure 4 plots the relationship between selection criteria versus a number of predictors in models. We observed that different criteria suggest different sized models that fit best. According to RSS criteria, five or six variable models fit the data better. Adjusted $R^{2}$ selected a six-variable model, while *BIC* and $C_{p}$ suggest four- and five-variable models, respectively.

**Figure 5:**
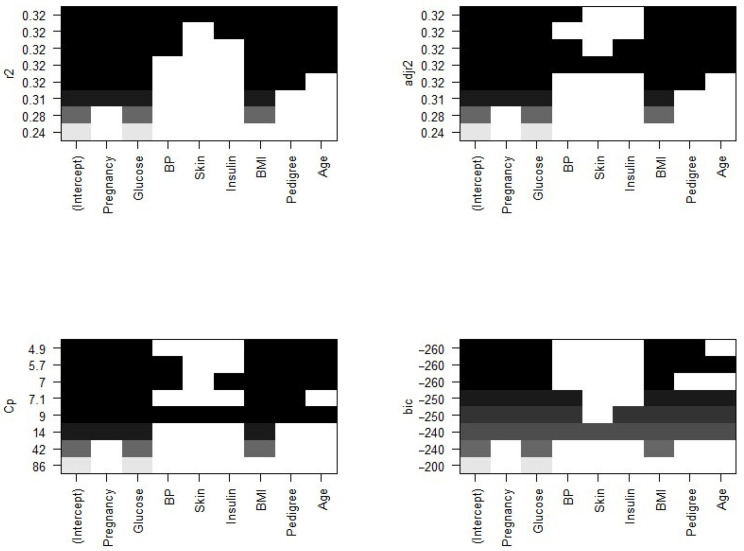
Significant predictors indicated by different criteria. Note: the variables BP and BMI are blood pressure and body mass index, whereas the metrics r2, Cp, adjr2, and bic represent R-squared, Mallows’ Cp, adjusted R-squared, and Bayesian information criteria, respectively.

Now, we need to know which of the predictors should be selected. According to the *BIC* criteria, there are only four predictors: pregnancy, glucose, BMI, and pedigree. The model with these four predictors has the minimum *BIC* value (results not shown). According to Mallows’ Cp criteria, we have five significant predictor variables: pregnancy, glucose, BMI, pedigree, and age. If we look at the adjusted $R^{2}$ plot, six variables, pregnancy, glucose, BP, BMI, pedigree, and age, are significant. The coefficients associated with the four predictors given by *BIC* and that for five predictors associated with the Mallows’ Cp criterion are given in Figure 5.

We further explored models suggested by the forward and backward stepwise selection method. For this, we used the *regsubsets()* function in R. The forward and backward method both agree on the five predictor variables, pregnancy, glucose, BMI, pedigree, and age, which is consistent with the other selection criteria to give the best fit model.

### 4.2. Classification Tree and Prediction Accuracy

**Figure 6:**
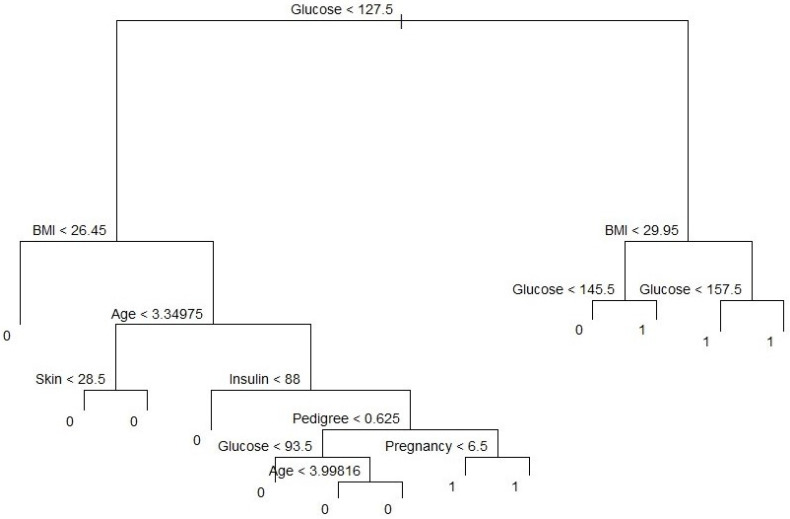
Classification tree with 13 terminal nodes. Note: BMI represents body mass index.

Our variable outcome, *diabetes*, is a binary numeric variable indexed by 1 and 0. We changed this variable into a qualitative variable to obtain a classification tree. Accordingly, we recoded variable labels so that “Yes” indicates the diabetes patient and “No” otherwise. Figure 5 shows the classification tree with thirteen terminal nodes and seven predictors. Deviance in the classification tree, as in James et al. [30], is given by

$$-2\sum_{m}\sum_{k}n_{mk}\log{\hat{p}}_{mk},$$

where $n_{mk}$ is the number of sample points in the mth terminal node that belong to the *kth* class. Small deviance indicates a good fit for the (training) data. The residual mean deviance measures the goodness of fit and is simply the deviance divided by $n-|T_{0}|$. Figure 5 shows a classification tree using all predictors available in the dataset. Glucose is the root node, suggesting that glucose is an important predictor of diabetes while BMI and age are also key variables in this method. Our results from this method are also in line with Wu et al. (2021), who indicated that glucose, BMI, and age were the top three predictors of diabetes. Residual mean deviance corresponding to Figure 6 is 601/755 = 0.796.

We consider the full-sized tree with thirteen terminal nodes to check the performance of different trees and subtrees. In order to evaluate the performance of a classification tree, we need to estimate the test error rate. For this, we split the dataset into a training set and a test set. The training dataset contains 75% of the observations and the test set contains the remaining 25% observations, which were assigned randomly. We built trees using the training set and evaluated their performance on the test data using the *predict()* function in statistical program R. The confusion matrix corresponding to the thirteen-node tree is given in Table 3.

The prediction accuracy rate of this thirteen-node tree is (102+40)/192=73.96%, meaning that this tree cannot predict accurately 26.07% of the time. We can check the performance of its subtrees by pruning for better predictive ability.

**Figure 7:**
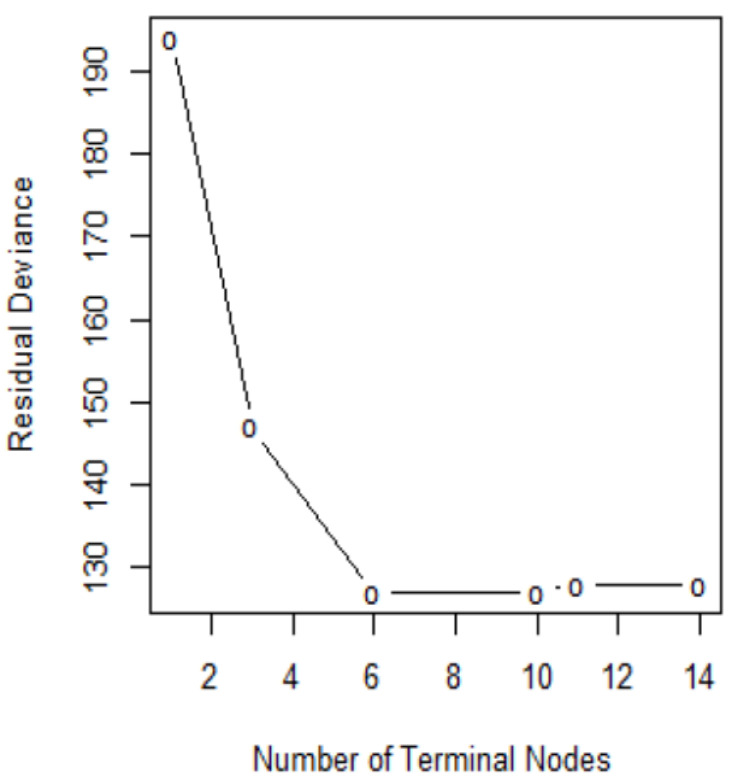
Pruning test.

In order to determine the optimal level of tree complexity, we employed the function *cv.tree()* in R. Cost complexity pruning was also used to select a sequence of trees for consideration. We applied the *prune.misclass()* function in R to prune the tree. We check for the performance of the trees with 14, 10, and 6 terminal nodes as suggested by the *cv.tree()function*. Figure 7 shows pruning plots which were determined by the size (the number of terminal nodes of tree) and α. The vertical axis represents residual deviance. The model with the lowest residual deviance is preferred, as shown in Table 4.

We observe that subtrees with ten and six terminal nodes have the lowest residual deviance; however, a tree with ten nodes may cause over-fitting. Thus, a six-node tree could be a better choice. The residual deviance for this tree is 127, and the optimal value of the tuning parameter is 3.25. The prediction accuracy corresponding to this six-terminal node tree is (106+37)/192=74.48% (Table 5). It means 74.48% of the test observations can be correctly classified with only four predictors: glucose, BMI, pedigree, and age. It also shows the improved accuracy compared to that of full-sized tree.

**Figure 8:**
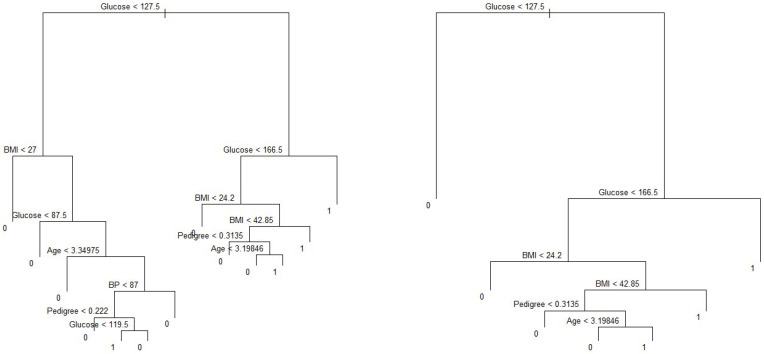
Pruned trees with ten and six internal nodes. Note: BMI and BP represent body mass index and blood pressure, respectively.

On the other hand, the predictors associated with the ten-node subtree are also the same—that is, glucose, BMI, pedigree and age, (Figure 8), with a prediction accuracy of (105+38)/192=74.48%, which is same as that obtained from a six-node subtree.

### 4.3. Proposed Diabetes Predictive Equation for Pima Indians

For the sake of completeness, we also calculated the prediction accuracy and cross-validation errors of the models proposed based on the logistic regression (Table 6). Model 6 has the highest prediction accuracy of 78.26% among all models considered in this study, and it has a classification error rate equal to 21.74%, which is better than that of the other candidate models.

To check the model’s predictive ability, we ran eight-fold cross-validation for the model, which gives a prediction error rate equal to 22.86%. This is slightly higher than that given in Table 7. Results provide evidence that model 6 with four interaction terms performs better than any combinations of covariates for the Pima Indian diabetes dataset. Thus, we propose the following model:

$$\begin{array}{ccc} Logit(\pi ) & = & -25.4+1.28*Pregnancy+0.16*Glucose+0.09*BMI\\ & & +1.73*Pedigree+4.4*Age-0.04*Insulin-0.32*Pregnancy\times \;Age\\ & & -0.03*Glucose\times Age-0.01*Pedigree\times Insulin+0.01*Age\times Insulin. \end{array}$$

## 5. Discussion

Diabetes has become one of the leading causes of human death in recent decades. The incidence of diabetes has been continuously increasing every year due to several reasons including eating habits, sedentary lifestyle, and prevalence of unhealthful foods. Diabetes prediction model can contribute to the decision-making process in clinical management. Knowing the potential risk factors and identifying individuals at high risk in the early stages may aid in diabetes prevention. A host of prediction models for diabetes have been developed and applied, out of which logistic regression [21] and a machine learning algorithm-based classification tree [20] are among the most popular methods. Habibi et al. [6] suggest that a simple machine learning algorithm, a classification tree, could be used to screen diabetes without using a laboratory. However, the validity of these models for different locations, populations with different diets, lifestyle, races, and genetic makeup is still unknown. Additionally, only a limited number of the reliable predictive equation has been suggested for Pima Indian Women. Their prediction performance and validity vary considerably. To fill this gap in the literature, this study used logistic regression and a classification tree from the Pima Indian dataset to identify the important factors for type 2 diabetes. We selected variables based on the goodness of fit test and model selection criteria such as *AIC*, *BIC*, and Mallows’ Cp. The decision trees were plotted, and the prediction accuracy and cross-validation error rate were calculated for the purpose of validation. Variables selected from the logistic regression and decision tree are very similar, suggesting that the variable identified helps predict diabetes and may be used as a decision tool.

A higher BMI, reduced insulin secretion and action, a family history of diabetes, blood pressure, smoking status, and pregnancy status are considered common risk factors for type 2 diabetes [33,34]. Our study shows five main predictors for diabetes are frequency of pregnancies in women, glucose, pedigree, BMI, and age. These variables were also used in previous studies to predict diabetes. For example, Bays et al. [35] reported that increased BMI was associated with an increased risk of diabetes mellitus. A study in Finland developed a diabetes risk score to predict diabetes and found that age, parental history of diabetes, BMI, high blood sugar level, and physical activities are among the predictors of diabetes [36]. People with higher glucose are more likely to develop diabetes. It may be because glucose is associated with insulin response [37]. Lyssenko et al. [33] reported that a family history of diabetes could double the risk of the disease. The pedigree provides a synthesis of the diabetes mellitus history in ancestors and the genetic relationship with the subject. It utilizes information from a person’s family history to predict how likely a subject can get diabetes. A higher BMI results in obesity, which could increase the fat content of the pancreas and might affect the function of pancreatic cells. Obesity could also lead to insulin resistance [38,39]. Age is a risk factor for the onset of diabetes. Pancreatic cells lead to the decline of glucose sensitivity and impaired insulin secretion with aging [40]. The validation shows that our model has a relatively good predictive performance. The prediction accuracy of 78% from our preferred specification is close to previous studies on diabetes risk factors. For example, Lyssenko et al. [33] report accuracy rates of 74% to 77% for two different locations in the study of diabetes risk factors. Zou et al. [20] predict diabetes with accuracy values of 77% and 81% for the Pima Indian and Luzhou datasets, respectively. As indicated by Wilson et al. [41], we also found that complex models are not necessary to predict diseases; instead, logistic regression and classification tree techniques can be equally useful in predicting diabetes. However, validation of the proposed model among different groups of the population should be carried out.

We acknowledge the limitations of our analysis. First, only a few predictors were considered to predict the risk of diabetes due to data limitations. Thus, our conclusion may not be generalizable to larger datasets with several predictors. Second, even the best predictive models and variable selection processes may yield different results according to location, type of dataset, and the algorithms used. Finally, we replaced missing values with medians of the respective variables which, although a common practice, could alter results. Future studies could incorporate several other risk factors such as genetic traits, gender, socio-economic status, physical activities, smoking, health information and attitude, food consumption, and spending to predicting diabetes in a more generalized population.

## 6. Conclusions

Identifying individuals at high risk of developing diabetes is a critical component of disease prevention and healthcare. This study presents a predictive equation of diabetes to provide a better understanding of risk factors that could assist in classifying high-risk individuals, make the diagnosis, and prevent and manage diabetes. Five critical variables identified in predicting type 2 diabetes are age, BMI, pedigree, glucose and frequency of pregnancies. We conclude that our proposed model has a prediction accuracy of 78.26% with a cross-validation error rate of 22.86%. As for the case of a classification tree, we would choose the tree with six nodes since it has the highest prediction accuracy (74.48%) than other possible subtrees. The results imply that if we control these five predictors by taking the necessary steps, it could lower type 2 diabetes prevalence. In addition, accurately predicting diabetes might help design interventions and implement health policies that may aid in disease prevention.

## Figures and Tables

**Table 1:** Descriptive statistics.

Variable	Definition	Mean	Std. dev.	Median
Pregnancy	Frequency of pregnancy	3.85	3.37	3.00
Glucose	Concentration of plasma glucose (mg/dL)	121.66	30.44	117.00
BP	Diastolic blood pressure (mm Hg)	72.39	12.10	72.00
Skin	Tricep skinfold thickness (mm)	29.11	8.79	29.00
Insulin	Two-hour serum insulin (mu U/ml)	140.67	86.38	125.00
BMI	Body mass index (kg/m2)	32.46	6.88	32.30
Pedigree	A pedigree function for diabetes	0.47	0.33	0.37
Age	Age (log (years))	33.24	11.76	29.00

**Table 2:** Predictive models for diabetes risk factors.

	Model 1	Model 2	Model 3	Model 4	Model 5	Model 6
Constant	$-0.62{\;}^{***}$	$-11.32{\;}^{***}$	$-11.43{\;}^{***}$	$-15.63{\;}^{***}$	$-23.92{\;}^{***}$	$-25.40{\;}^{***}$
	(0.08)	(1.33)	(1.32)	(1.96)	(5.35)	(5.62)
Pregancy		$0.11{\;}^{**}$	$0.10{\;}^{**}$	$1.29{\;}^{**}$	$1.29{\;}^{***}$	$1.28{\;}^{**}$
		(0.03)	(0.03)	(0.39)	(0.39)	(0.40)
Glucose		$0.04{\;}^{***}$	$0.04{\;}^{***}$	$0.04{\;}^{***}$	$0.10{\;}^{**}$	$0.16{\;}^{***}$
		(0.00)	(0.00)	(0.00)	(0.04)	(0.04)
BP		−0.01				
		(0.01)				
Skin		0.00				
		(0.01)				
Insulin		−0.00				$-0.04{\;}^{**}$
		(0.00)				(0.01)
BMI		$0.10{\;}^{***}$	$0.09{\;}^{***}$	$0.09{\;}^{***}$	$0.09{\;}^{***}$	$0.09{\;}^{***}$
		(0.02)	(0.01)	(0.01)	(0.02)	(0.02)
Pedigree		$0.86{\;}^{**}$	$0.86{\;}^{**}$	$0.90{\;}^{**}$	$0.87{\;}^{**}$	$1.73{\;}^{***}$
		(0.30)	(0.30)	(0.30)	(0.30)	(0.45)
Age		$0.84{\;}^{*}$	$0.73{\;}^{*}$	$1.89{\;}^{***}$	$4.27{\;}^{**}$	$4.40{\;}^{**}$
		(0.37)	(0.35)	(0.52)	(1.51)	(1.58)
Pregancy × Age				$-0.33{\;}^{**}$	$-0.33{\;}^{**}$	$-0.32{\;}^{**}$
				(0.11)	(0.11)	(0.11)
Glucose × Age					−0.02	$-0.03{\;}^{**}$
					(0.01)	(0.01)
Pedigree × Insulin						$-0.01{\;}^{**}$
						(0.00)
Age × Insulin						$0.01{\;}^{**}$
						(0.00)
*AIC*	995.48	727.55	724.13	716.70	715.90	701.87
*BIC*	1000.13	769.34	752.00	749.20	753.05	752.95
Log Likelihood	−496.74	−354.77	−356.07	−351.35	−349.95	−339.93
Deviance	993.48	709.55	712.13	702.70	699.90	679.87
$R^{2}$	0.00	0.43	0.42	0.43	0.44	0.46
N	768	768	768	768	768	768

Note: Figures in parentheses indicate standard errors. ${}^{***}\;p<0.001$; ${}^{**}\;p<0.01$; ${}^{*}\;p<0.05$. BP, BMI and Skin represent blood pressure, body mass index and tricep skinfold thickness, respectively.

**Table 3:** Confusion matrix on test dataset.

	Predicted		
Actual		No	Yes
	No	102	27
	Yes	23	40

**Table 4:** Relationship between terminal nodes and residual deviance.

Parameter	(1)	(2)	(3)	(4)	(5)	(6)
Size	14	11	10	6	3	1
Residual deviance	128	128	127	127	147	194
α	−∞	0	2	3.25	7.67	30

**Table 5:** Confusion matrix for a tree with six terminal nodes.

	Predicted		
Actual		No	Yes
	No	106	29
	Yes	20	37

**Table 6:** Summary of prediction accuracy and validation errors of potential predictive models.

Accuracy/Error	Model 2	Model 3	Model 4	Model 5	Model 6
Prediction accuracy	77:73%	74.48%	76.43%	76.17%	78.26%
Classification error	22.27%	25.52%	23.57%	23.83 %	21.74%
Cross validation error	22.65%	25.14%	23.69%	22.86%	22.86%

**Table 7:** Confusion matrix of proposed model.

	Predicted		
Actual		No	Yes
	No	442	58
	Yes	109	159

## Data Availability

Data are available in a publicly accessible repository that does not issue DOIs. Publicly available datasets were analyzed in this study. This data can be found here: https://www.kaggle.com/uciml/pima-indians-diabetes-database.

## Abbreviations

The following abbreviations are used in this manuscript:

mg/dL	milligrams per deciliter
mm	milliliter
Kg	kilogram
mm Hg	millimeters of mercury
BMI	Body mass index
DPF	Pedigree function for diabetes
BP	Blood pressure
*AIC*	Akaike’s information criteria
*BIC*	Bayesian information criteria
CDC	Centers for disease control and prevention

## References

[1] Cho N., Shaw J., Karuranga S., Huang Y., da Rocha Fernandes J., Ohlrogge A., Malanda B. (2018). IDF Diabetes Atlas: Global estimates of diabetes prevalence for 2017 and projections for 2045. Diabetes Res. Clin. Pract..

[2] CDC (2020). Centers for Disease Control and Prevention and Others.

[3] Krasteva A., Panov V., Krasteva A., Kisselova A., Krastev Z. (2011). Oral cavity and systemic diseases—Diabetes mellitus. Biotechnol. Biotechnol. Equip..

[4] Alghamdi M., Al-Mallah M., Keteyian S., Brawner C., Ehrman J., Sakr S. (2017). Predicting diabetes mellitus using SMOTE and ensemble machine learning approach: The Henry Ford Exercise Testing (FIT) project. PLoS ONE.

[5] Nguyen B.P., Pham H.N., Tran H., Nghiem N., Nguyen Q.H., Do T.T., Tran C.T., Simpson C.R. (2019). Predicting the onset of type 2 diabetes using wide and deep learning with electronic health records. Comput. Methods Programs Biomed..

[6] Habibi S., Ahmadi M., Alizadeh S. (2015). Type 2 diabetes mellitus screening and risk factors using decision tree: Results of data mining. Glob. J. Health Sci..

[7] Ryden L., Standl E., Bartnik M., Van den Berghe G., Betteridge J., De Boer M.J., Cosentino F., Jönsson B., Laakso M., Malmberg K. (2007). Guidelines on diabetes, pre-diabetes, and cardiovascular diseases: Executive summary: The Task Force on Diabetes and Cardiovascular Diseases of the European Society of Cardiology (ESC) and of the European Association for the Study of Diabetes (EASD). Eur. Heart J..

[8] Tuso P. (2014). Prediabetes and lifestyle modification: Time to prevent a preventable disease. Perm. J..

[9] IDF Clinical Guidelines Task Force (2006). Global Guideline for Type 2 Diabetes: Recommendations for standard, comprehensive, and minimal care. Diabet. Med..

[10] Gregg E.W., Geiss L.S., Saaddine J., Fagot-Campagna A., Beckles G., Parker C., Visscher W., Hartwell T., Liburd L., Narayan K.V. (2001). Use of diabetes preventive care and complications risk in two African-American communities. Am. J. Prev. Med..

[11] Knowler W.C., Barrett-Connor E., Fowler S.E., Hamman R.F., Lachin J.M., Walker E.A., Nathan D.M., Diabetes Prevention Program Research Group (2002). Reduction in the incidence of type 2 diabetes with lifestyle intervention or metformin. N. Engl. J. Med..

[12] Wild S., Roglic G., Green A., Sicree R., King H. (2004). Global prevalence of diabetes: Estimates for the year 2000 and projections for 2030. Diabetes Care.

[13] Engelgau M.M., Narayan K., Herman W.H. (2000). Screening for type 2 diabetes. Diabetes Care.

[14] Rolka D.B., Narayan K.V., Thompson T.J., Goldman D., Lindenmayer J., Alich K., Bacall D., Benjamin E.M., Lamb B., Stuart D.O. (2001). Performance of recommended screening tests for undiagnosed diabetes and dysglycemia. Diabetes Care.

[15] Schwarz P.E., Li J., Lindstrom J., Tuomilehto J. (2009). Tools for predicting the risk of type 2 diabetes in daily practice. Horm. Metab. Res..

[16] Yu W., Liu T., Valdez R., Gwinn M., Khoury M.J. (2010). Application of support vector machine modeling for prediction of common diseases: The case of diabetes and pre-diabetes. BMC Med. Inform. Decis. Mak..

[17] Naz H., Ahuja S. (2020). Deep learning approach for diabetes prediction using PIMA Indian dataset. J. Diabetes Metab. Disord..

[18] Heikes K.E., Eddy D.M., Arondekar B., Schlessinger L. (2008). Diabetes Risk Calculator: A simple tool for detecting undiagnosed diabetes and pre-diabetes. Diabetes Care.

[19] Razavian N., Blecker S., Schmidt A.M., Smith-McLallen A., Nigam S., Sontag D. (2015). Population-level prediction of type 2 diabetes from claims data and analysis of risk factors. Big Data.

[20] Zou Q., Qu K., Luo Y., Yin D., Ju Y., Tang H. (2018). Predicting diabetes mellitus with machine learning techniques. Front. Genet..

[21] Christodoulou E., Ma J., Collins G.S., Steyerberg E.W., Verbakel J.Y., Van Calster B. (2019). A systematic review shows no performance benefit of machine learning over logistic regression for clinical prediction models. J. Clin. Epidemiol..

[22] Nusinovici S., Tham Y.C., Yan M.Y.C., Ting D.S.W., Li J., Sabanayagam C., Wong T.Y., Cheng C.Y. (2020). Logistic regression was as good as machine learning for predicting major chronic diseases. J. Clin. Epidemiol..

[23] Anderson A.E., Kerr W.T., Thames A., Li T., Xiao J., Cohen M.S. (2016). Electronic health record phenotyping improves detection and screening of type 2 diabetes in the general United States population: A cross-sectional, unselected, retrospective study. J. Biomed. Inform..

[24] Collins G.S., Mallett S., Omar O., Yu L.M. (2011). Developing risk prediction models for type 2 diabetes: A systematic review of methodology and reporting. BMC Med..

[25] Kalil A.C., Mattei J., Florescu D.F., Sun J., Kalil R.S. (2010). Recommendations for the assessment and reporting of multivariable logistic regression in transplantation literature. Am. J. Transplant..

[26] Mikolajczyk R.T., DiSilvesto A., Zhang J. (2008). Evaluation of logistic regression reporting in current obstetrics and gynecology literature. Obstet. Gynecol..

[27] Bennett P., Burch T., Miller M. (1971). Diabetes mellitus in American (Pima) indians. Lancet.

[28] Ravussin E., Valencia M.E., Esparza J., Bennett P.H., Schulz L.O. (1994). Effects of a traditional lifestyle on obesity in Pima Indians. Diabetes Care.

[29] R Core Team (2021). R: A Language and Environment for Statistical Computing.

[30] James G., Witten D., Hastie T., Tibshirani R. (2013). An Introduction to Statistical Learning.

[31] Kavakiotis I., Tsave O., Salifoglou A., Maglaveras N., Vlahavas I., Chouvarda I. (2017). Machine learning and data mining methods in diabetes research. Comput. Struct. Biotechnol. J..

[32] Brieman L., Friedman J., Olshen R., Stone C. (2017). Classification and Regression Trees.

[33] Lyssenko V., Jonsson A., Almgren P., Pulizzi N., Isomaa B., Tuomi T., Berglund G., Altshuler D., Nilsson P., Groop L. (2008). Clinical risk factors, DNA variants, and the development of type 2 diabetes. N. Engl. J. Med..

[34] Tirosh A., Shai I., Tekes-Manova D., Israeli E., Pereg D., Shochat T., Kochba I., Rudich A. (2005). Normal fasting plasma glucose levels and type 2 diabetes in young men. N. Engl. J. Med..

[35] Bays H.E., Chapman R., Grandy S., Group S.I. (2007). The relationship of body mass index to diabetes mellitus, hypertension and dyslipidaemia: Comparison of data from two national surveys. Int. J. Clin. Pract..

[36] Lindström J., Tuomilehto J. (2003). The diabetes risk score: A practical tool to predict type 2 diabetes risk. Diabetes Care.

[37] Lorenzo C., Wagenknecht L.E., Hanley A.J., Rewers M.J., Karter A.J., Haffner S.M. (2010). A1C between 5.7 and 6.4% as a marker for identifying pre-diabetes, insulin sensitivity and secretion, and cardiovascular risk factors: The Insulin Resistance Atherosclerosis Study (IRAS). Diabetes Care.

[38] Barazzoni R., Cappellari G.G., Ragni M., Nisoli E. (2018). Insulin resistance in obesity: An overview of fundamental alterations. Eat. Weight Disord.-Stud. Anorexia Bulim. Obes..

[39] Wu Y., Hu H., Cai J., Chen R., Zuo X., Cheng H., Yan D. (2020). A prediction nomogram for the 3-year risk of incident diabetes among Chinese adults. Sci. Rep..

[40] De Tata V. (2014). Age-related impairment of pancreatic Beta-cell function: Pathophysiological and cellular mechanisms. Front. Endocrinol..

[41] Wilson P.W., Meigs J.B., Sullivan L., Fox C.S., Nathan D.M., D’Agostino R.B. (2007). Prediction of incident diabetes mellitus in middle-aged adults: The Framingham Offspring Study. Arch. Intern. Med..

